# Hypertonic Stress Induces VEGF Production in Human Colon Cancer Cell Line Caco-2: Inhibitory Role of Autocrine PGE_2_

**DOI:** 10.1371/journal.pone.0025193

**Published:** 2011-09-28

**Authors:** Luciana B. Gentile, Bruno Piva, Bruno L. Diaz

**Affiliations:** 1 Divisão de Biologia Celular, Coordenação de Pesquisa, Instituto Nacional de Câncer, Rio de Janeiro, Rio de Janeiro, Brasil; 2 Programa de Pós-Graduação em Ciências Morfológicas, Instituto de Ciências Biomédicas, Universidade Federal do Rio de Janeiro, Rio de Janeiro, Rio de Janeiro, Brasil; 3 Programa de Imunobiologia, Instituto de Biofísica, Universidade Federal do Rio de Janeiro, Rio de Janeiro, Rio de Janeiro, Brasil; Chinese University of Hong Kong, Hong Kong

## Abstract

Vascular Endothelial Growth Factor (VEGF) is a major regulator of angiogenesis. VEGF expression is up regulated in response to micro-environmental cues related to poor blood supply such as hypoxia. However, regulation of VEGF expression in cancer cells is not limited to the stress response due to increased volume of the tumor mass. Lipid mediators in particular arachidonic acid-derived prostaglandin (PG)E_2_ are regulators of VEGF expression and angiogenesis in colon cancer. In addition, increased osmolarity that is generated during colonic water absorption and feces consolidation seems to activate colon cancer cells and promote PGE_2_ generation. Such physiological stimulation may provide signaling for cancer promotion. Here we investigated the effect of exposure to a hypertonic medium, to emulate colonic environment, on VEGF production by colon cancer cells. The role of concomitant PGE_2_ generation and MAPK activation was addressed by specific pharmacological inhibition. Human colon cancer cell line Caco-2 exposed to a hypertonic environment responded with marked VEGF and PGE_2_ production. VEGF production was inhibited by selective inhibitors of ERK 1/2 and p38 MAPK pathways. To address the regulatory role of PGE_2_ on VEGF production, Caco-2 cells were treated with cPLA_2_ (ATK) and COX-2 (NS-398) inhibitors, that completely block PGE_2_ generation. The Caco-2 cells were also treated with a non selective PGE_2_ receptor antagonist. Each treatment significantly increased the hypertonic stress-induced VEGF production. Moreover, addition of PGE_2_ or selective EP_2_ receptor agonist to activated Caco-2 cells inhibited VEGF production. The autocrine inhibitory role for PGE_2_ appears to be selective to hypertonic environment since VEGF production induced by exposure to CoCl_2_ was decreased by inhibition of concomitant PGE_2_ generation. Our results indicated that hypertonicity stimulates VEGF production in colon cancer cell lines. Also PGE_2_ plays an inhibitory role on VEGF production by Caco-2 cells exposed to hyperosmotic stress through EP_2_ activation.

## Introduction

Formation of new blood vessels from pre-existing vasculature is a central process in the development of most tumors especially solid ones. This process is called angiogenesis and is regulated by the balance of negative and positive biochemical signals. The newly formed blood vessels are responsible for supplying oxygen and nutrients for the growing tumor mass and a route for dissemination of metastatic cancerous cells. VEGF is the most prominent positive regulator of angiogenesis due to its ability to recruit endothelial cells to hypoxic sites and to stimulate the proliferation of this cellular type, promoting the differentiation of vascular structures [1]. VEGF expression correlates positively with negative outcome in cancer patients. In colon cancer, expression of VEGF correlates with increased metastatic potential [2], while expression of its receptor is a marker of shorter post-operative survival [3].

VEGF expression is up regulated in response to micro-environmental cues related to poor blood supply such as hypoxia [4], acidosis [5] and low nutrient levels [6]. In tumors, decreased levels of O_2_ leads to HIF-1α stabilization, a subunit of the transcriptional factor HIF-1, and subsequent transcriptional activation of genes presenting a hypoxia-responsive element (HRE) in their promoters, such as VEGF. However, VEGF expression regulation in cancer cells is not limited to the stress response due to the increased volume of the tumor mass. Several other factors have been shown to induce VEGF such as reactive oxygen species [7]–[9], growth factors [10], [11], cytokines [12], and lipid mediators [13]–[16]. Arachidonic acid-derived prostaglandin (PG)E_2_ is a major regulator of VEGF expression and angiogenesis in several different cancer types and in colon cancer in particular. Exogenous PGE_2_ induces HIF-1α stabilization [13] and VEGF expression [17] in colon cancer cell lines. VEGF and COX-2 expression and tumor angiogenesis are positively correlated in colon cancer samples [18]–[20].

However, hypoxia is not the only external stress stimulus which activates cellular responses in colon cancer. The continuously changing contents of intestinal lumen expose normal and cancerous epithelial cells to a myriad of stimuli. Such physiological stimulation may provide signaling for cancer promotion. In fact, increased osmolarity that is generated during the process of colonic water absorption and feces consolidation [21]–[23] appears to activate colon cancer cells and promote COX-2 expression and PGE_2_ generation but does not activate normal intestinal cells [24]. Our aim in this study was to determine the effect of hypertonic stress on VEGF production by Caco-2 colon cancer cell line. The potential role of autocrine PGE_2_ and MAPK signaling pathways in the modulation of VEGF generation was also analyzed.

## Methods

### Reagents

Sodium chloride (NaCl) was obtained from Sigma Chemical Co. (St. Louis, MO) and dissolved in sterile water for a stock solution at 2 M concentration. The iPLA_2_ inhibitor, Bromoenol lactone (BEL), the cPLA_2_/iPLA_2_ inhibitor, Arachidonyl Trifluoromethyl Ketone (ATK), and Prostaglandin E_2_ were purchased from Cayman Chemical Co. (Ann Arbor, MI) and diluted in ethanol accordingly to manufacturer's instructions. The inhibitors for COX-2, NS-398 (Cayman); p38, SB202190; JNK, SP600125; MEK1/2, U0126 (all from BIOMOL, Plymouth Meeting, PA) were diluted in DMSO (Sigma). Monoclonal antibodies for immunoblot assays were anti-COX-2 IgG mouse (clone 33) from BD Transduction Laboratories and anti-GAPDH (clone 6C5) IgG mouse from Santa Cruz Biotechnology (Santa Cruz, CA), diluted at 0.003 µg/mL. The goat HRP-linked secondary antibody anti-mouse IgG from Santa Cruz Biotechnology was used at 0.1 µg/mL.

### Cell culture and treatments

Caco-2 (ATCC HTB-37, gift of Dr. José Morgado Díaz, Instituto Nacional de Câncer, Brazil) cell line was maintained in Dulbecco Modified Eagle medium (DMEM) supplemented with 10% fetal bovine serum (FBS), 44 mM NaHCO_3_, 1 mM NaH_2_PO_4_.H_2_O, 1 mM sodium pyruvate, 10 mM HEPES, MEM vitamins solution, MEM essential and non-essential amino acids solution, 2 mM L-glutamine, 55 µM β-mercaptoethanol, 100 U/mL penicillin, and 100 µg/mL streptomycin (all cell culture reagents from Invitrogen). IEC-6 cell line (Rio de Janeiro Cell Bank, Brazil) was maintained in DMEM supplemented with 5% FBS and 100 U/mL penicillin, and 100 µg/mL streptomycin. Cells were maintained in culture flasks (cell growth surface area 25, 75 and 150 cm^2^). Cells were collected by 0.25% trypsin and 0.38 g/L EDTA in HBSS without Ca^++^ and Mg^++^ and 5×10^5^ cells/well were plated in 6-well flat-bottom plates (area of 9.03 cm^2^/well, Techno Plastic Products, Switzerland). 1.9 mL of fresh supplemented DMEM culture medium was added to culture wells with or without pharmacological inhibitors and cells were incubated for 15 min (30 min for MAP kinases inhibitors) at 37°C. All cells received the same amount of vehicle, therefore, the final concentration was below 0.1% of DMSO or ethanol and did not modify cell activation. Cells were stimulated with the addition of 0–100 µL of 2 M NaCl solution. DMEM was added to the well to complete final volume of 2 mL. Final osmolarity of the medium after addition of 100 mM NaCl was approximately 540 mOsm as compared to 367 mOsm of isosmotic medium. Medium osmolarity was empirically determined by freezing method using an osmometer (Advanced Instruments Inc., Norwood, MA). To minimize variation in the kinetic experiments the total time in culture after plating was the same for every time point analyzed.

### Determination of PGE_2_ and VEGF on supernatants

The PGE_2_ production by Caco-2 cell line was determined by EIA in culture supernatant accordingly to manufacturer's instructions (Cayman) and as described before [25]. Briefly, culture medium was collected 24 h after cellular activation by hypertonic stress and centrifuged at 250×g for 5 min to remove floating cells and frozen at −70°C. PGE_2_ levels were assayed using a monoclonal antibody PGE_2_ EIA Kit. After development plate was read at 405 nm in a plate reader (Spectra Max 190, Molecular Devices, Sunnyvale, CA). The VEGF production by Caco-2 cell line was determined by ELISA in culture supernatant accordingly to manufacturer's instructions (R&D Systems, UK). Briefly, culture medium was collected 24 h after cellular activation by hypertonic stress and centrifuged at 250×g for 5 min to remove floating cells and frozen at −70°C. VEGF levels were assayed using a human VEGF DuoSet (R&D Systems, UK). After development plate was read at 450 nm in a plate reader (Spectra Max 190, Molecular Devices, Sunnyvale, CA).

### Immunoblot analysis

Cells were collected with a cell scraper (COSTAR) and 100 µL of 10% SDS was added per well of the 6-well cell culture plate. 100 µL of 2× loading buffer (1.4 M β-mercaptoethanol, 184 mM Tris base, 80 µM Bromophenol blue, 3% glycerol, 8% SDS, pH 6.8) was added to the cell lysate. Cellular lysates were immediately heated at 100°C for 5 minutes prior to sonication at 30% of amplitude and 30 J of energy in a high intensity ultra-sonic processor. Samples were resolved by electrophoresis on a SDS-PAGE 10% polyacrylamide gel at 29 mA/gel for 1 h. Separated proteins were transferred to nitrocellulose membranes (Santa Cruz) and blocked in 5% non-fat dry milk in 1× TBS (Tris 10 mM; NaCl 150 mM pH 7,4) for 12 h for COX-2 labeling, or for 2 h for all other antibodies at room temperature. After washing, membranes were incubated with primary antibodies diluted in TTBS (TBS with 0.2% Tween 20) and 0.05% sodium azide for 2 hours at room temperature for COX-2 or 12 hours at 4°C for other antibodies. After secondary labeling with HRP-linked anti-IgG antibodies, proteins were analyzed using ECL Western Blotting Analysis System (Amersham Biosciences).

### Statistical analysis

Data are expressed as mean ± standard error of the mean (SEM) of experiments performed in triplicates. Graphs and western blots shown are representative of at least three independent experiments. Multiple comparisons among groups were performed by one-way ANOVA followed by Bonferroni's or Dunnett's test (Prism version 4.03, Graphpad Software, Inc. La Jolla, CA). The symbols ^+^ and * represent *p* values<0.05 when compared to control non-stimulated group or hypertonic stress/CoCl_2_-stimulated group respectively.

## Results

### Hypertonic stress induces VEGF production by Caco-2 and PGE_2_ modulates the angiogenic factor production in this environmental stress

**Figure 1:**
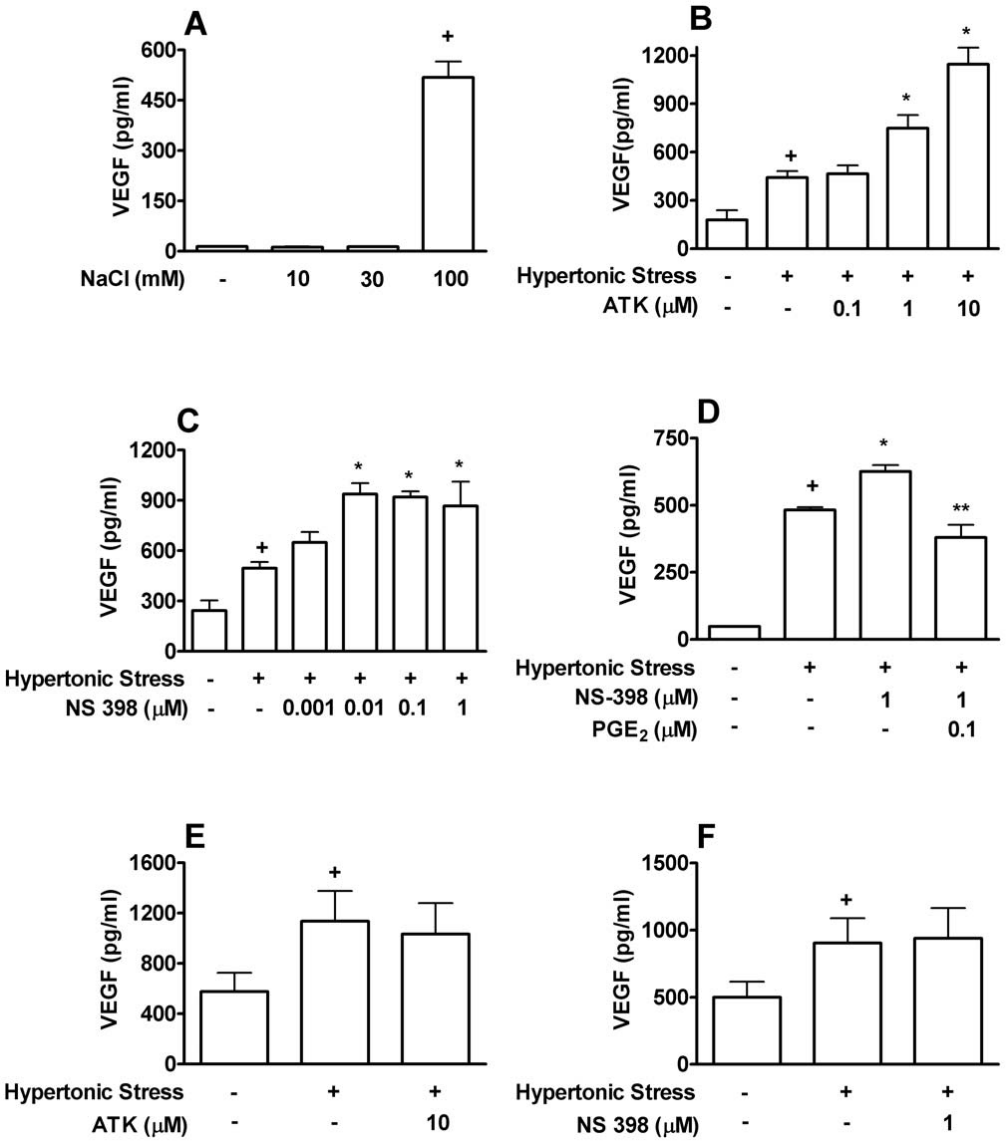
Endogenous PGE_2_ modulates VEGF production by hypertonic stress-stimulated Caco-2 cells. (**A**) Caco-2 cells were stimulated with 10–100 mM of NaCl during 24 h before VEGF production analysis. VEGF production was determined by ELISA in supernatants of Caco-2 cells stimulated with hypertonic stress (100 mM NaCl) during 24 h after pre-treatment with inhibitors of cPLA_2_, ATK (**B**); COX-2, NS-398 (**C**) or PGE_2_ (**D**). HCT116 cells were stimulated with 100 mM of NaCl during 24 h after pre-treatment with inhibitors of cPLA_2_, ATK (**E**); COX-2, NS-398 (**F**). +, * *p<0.05*, to non-stimulated cells or stimulated cells, respectively. ** *p<0.05*, when compared to NS-398-treated cells. Graph bars show means ± SEM from triplicate samples.

Hypertonic stress stimulates the VEGF production by Caco-2 after 24 hours of activation (Figure 1A) following the same dose response and time course (data not shown) of PGE_2_ generation under the same stimulatory conditions [24]. As PGE_2_ has been described to play a role in angiogenesis and VEGF production we determined whether PGE_2_ was regulating VEGF production by Caco-2 during the stimulation with hypertonic medium. Inhibiton of PGE_2_ production by treatment with cPLA_2_ (ATK) (Figure 1B) or COX-2 (NS-398) (Figure 1C) inhibitors increased VEGF production. This phenomenon was reversed by the addition of PGE_2_ to the cell culture medium (Figure 1D) indicating a specific role for PGE_2_.

To verify whether inhibition of VEGF production by Caco-2 stimulated with hypertonic stress was a consequence of PGE_2_ action and not a consequence of the nonespecific action of pharmacological drugs used in the experiments, we performed the same experiment in HCT116, a colon cancer cell line that does not express COX-2 and produces no detectable levels of PGE_2_ under hypertonic stress. We did not observe any changes in the VEGF production, excluding the possibility of interference of the pharmacological inhibitors (Figures 1E and F). Those results showed that PGE_2_ produced by Caco-2 activated by hypertonic stress has an autocrine inhibitory action on VEGF production.

### EP_2_ receptor plays a role in the regulation of VEGF production through PGE_2_ in Caco-2 stimulated with hypertonic stress

**Figure 2:**
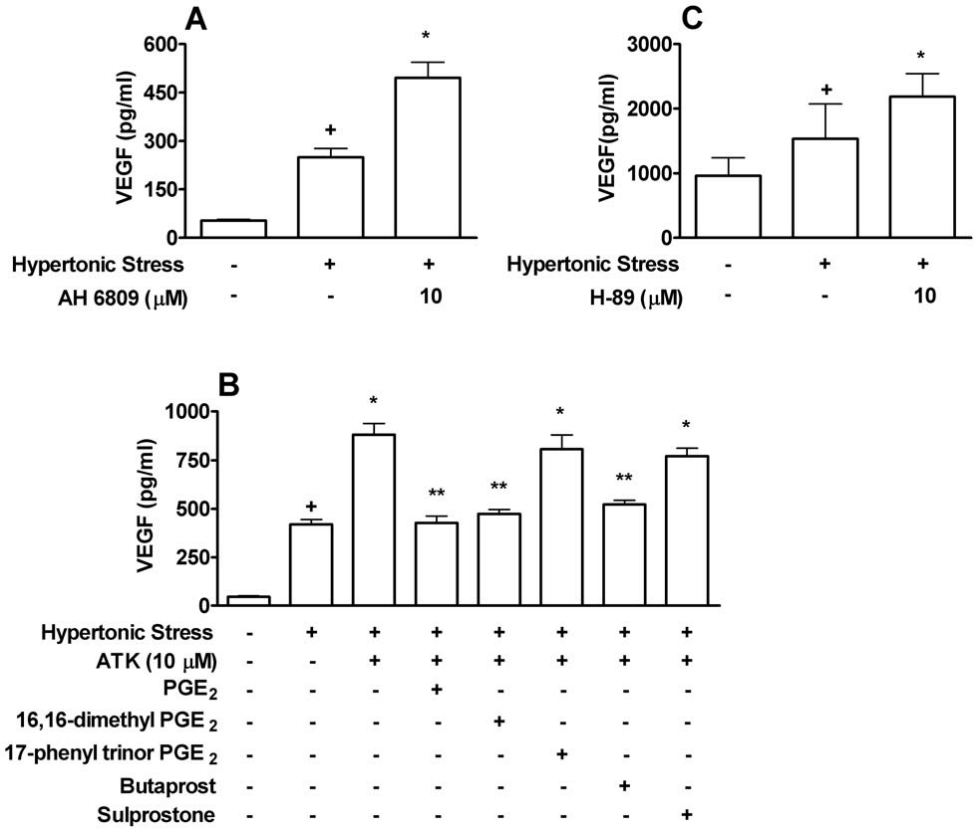
EP_2_ receptor plays a role in endogenous PGE_2_ regulation of VEGF production by Caco-2 stimulated with hypertonic stress. (**A**) Caco-2 cells were stimulated by hypertonic stress (100 mM NaCl) during 24 h after pre-treatment with EP and DP receptors antagonist, AH 6809 (**A**); with inhibitor of cPLA_2_, ATK (10 µM); PGE_2_; EP receptors agonist, 16,16-dimethyl Prostaglandin E_2_; EP1 and EP3 receptors agonist, 17-phenyl trinor Prostaglandin E_2_; EP2 receptor agonist, butaprost and EP3 receptor agonist, sulprostone (**B**); or with PKA inhibitor, H-89. PGE_2_ and its analogs were used at 0.1 µM. VEGF production was determined by ELISA in supernatants of Caco-2 cells. +, * *p<0.05*, when compared to non-stimulated cells or stimulated cells, respectively. ** *p<0.05*, when compared to ATK-treated cells. Graph bars show means ± SEM from triplicate samples.

To determine what is the mechanism of action of PGE_2_ in the regulation of VEGF production in hypertonic stress, Caco-2 cells were activated with hypertonic medium during 24 hours and treated with AH6809, an EP receptor antagonist. We verified the inhibition of EP receptor signaling caused an increase of VEGF production (Figure 2A). Then, to identify which specific receptor is involved in this phenomenon Caco-2 were stimulated with hypertonic medium and treated with ATK and EP receptors agonists. The cPLA_2_ inhibitor (ATK) removed the interference of PGE_2_ produced by colon cancer cells and EP receptors were activated only by exogenously added agonists. Accordingly, figure 2B shows that treatment with ATK increases VEGF production and when PGE_2_ or 16,16-dimetil PGE_2_, a pan EP receptor agonist, were added to the medium this effect was reversed, reinforcing that EP receptor activation has an inhibitory effect on VEGF production. Increase in VEGF production by inhibition of endogenous PGE_2_ was also reversed by butaprost, a specific EP2 receptor agonist (Figure 2B). Activation with EP1, 17-phenyl-trino-PGE_2_, or EP3, Sulprostone, agonists had no effect on increased VEGF production. Since EP4 expression seems to be absent in Caco-2 cells [26], our results indicate that endogenous PGE_2_ modulates VEGF production induced by hypertonic stress through activation of EP2.

EP2 is a rhodopsin type receptor coupled to Gs and mediates increases in camp [27]. Thus we tested if PKA played a role in PGE_2_ effects mediated through EP2 during hypertonic stress. Inhibition of PKA by H-89 increased VEGF production by Caco-2 cells exposed to hypertonic medium (Figure 2C). Reinforcing the potential inhibitory pathway involving PGE_2_-EP2-cAMP-PKA.

### Role of endocrine PGE_2_ on CoCl_2_-induced VEGF production

**Figure 3:**
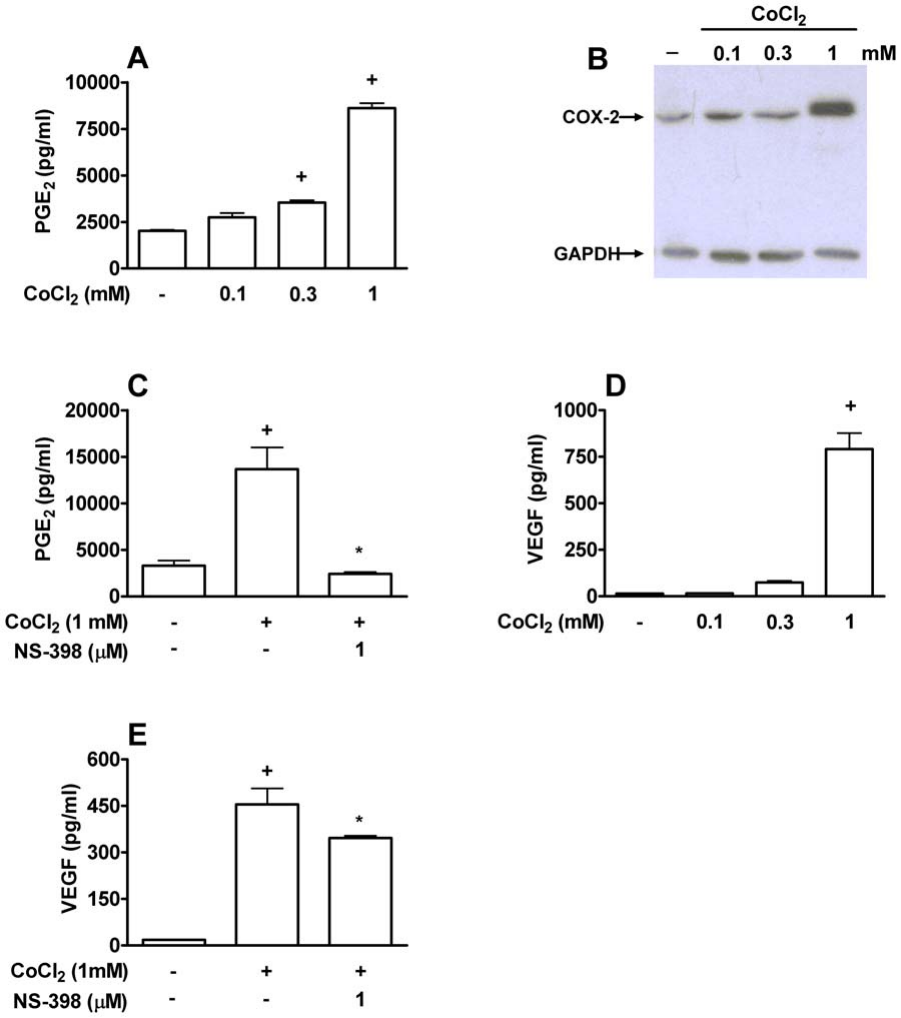
PGE_2_ stimulates VEGF production by Caco-2 cells activated with CoCl_2_. Caco-2 cells were stimulated with 0.1–1 mM of CoCl_2_ during 24 h before PGE_2_ and VEGF production (**A** and **D**, respectively) and COX-2 protein expression (**B**) analysis. PGE_2_ and VEGF production by Caco-2 cells stimulated with 1 mM of CoCl_2_ during 24 h after pre-treatment with inhibitor of COX-2, NS-398 (**C** and **E**, respectively). VEGF production by Caco-2 cells stimulated with 0.1–1 mM of CoCl_2_ during 24 h (**D**). PGE_2_ and VEGF production were determined by ELISA in supernatants of Caco-2 cells. COX-2 and GAPDH expression in cell pellets was analyzed by Western blotting. +, * *p<0.05*, when compared to non-stimulated cells or stimulated cells, respectively. Graph bars show means ± SEM from triplicate samples.

We also verified whether PGE_2_ had a role in the regulation of VEGF production under a standard simulatory condition. To activate the Hypoxia-Induced Factor (HIF) pathway and simulate hypoxia, Caco-2 cells were activated with CoCl_2_. After 24 hours of stimulation with CoCl_2_, Caco-2 presented marked production of PGE_2_ (Figure 3A) and increased expression of COX-2 (Figure 3B). Further experiments were performed after additon of 1 mM of CoCl_2_ to the medium after 24 hours of activation. Moreover, CoCl_2_-stimulated PGE_2_ generation is dependent on COX-2 as it was completely inhibited by NS-398 (Figure 3C).

Similar to hypertonic stress stimulation, CoCl_2_-activated cell also produced VEGF (Figure 3D). However, autocrine PGE_2_ appears to have an opposite effect in this condition since NS-398 treatment inhibited VEGF production (Figure 3E). Those results indicate that PGE_2_ has a stimulatory autocrine role on VEGF production in CoCl_2_ activation.

### Role of MAPKs in VEGF production by Caco-2 cells

**Figure 4:**
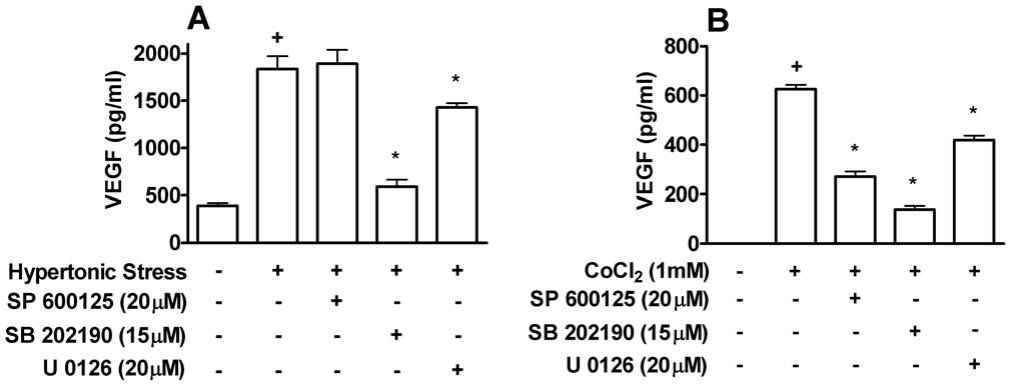
Role of MAPKs in VEGF production by Caco-2 cells. Inhibitors of JNK, SP600125; p38, SB202190; and MEK 1/2, U0126 were added before stimulation with hypertonic stress (100 mM NaCl) (**A**) or 1 mM CoCl_2_ (**B**) for 24 h. Caco-2 cells were pretreated with 1 µM of NS-398 to prevent endogenous PGE_2_ production in all samples. VEGF production was determined by ELISA in supernatants of Caco-2 cells. +, * *p<0.05*, when compared to non-stimulated cells or stimulated cells, respectively. Graph bars show means ± SEM from triplicate samples.

To determine if the VEGF production was differentially regulated in Caco-2 cells beyond the potential autocrine role of PGE_2_, we turned to the identification of MAPK pathways involved. Since we have shown before a role for ERK 1/2, JNK and p38 in PGE_2_ generation [24] and to avoid the potential problem this may pose to interpret the results, experiments were performed in the presence of 1 µM of NS-398. Pharmacological inhibition of ERK 1/2 and p38 pathways indicated a common role in activation by either hypertonic stress or CoCl_2_ (Figures 4A and B). JNK role was more restricted, as SP 600125 markedly inhibited VEGF production induced by CoCl_2_ activation while it did not affect VEGF production induced by hypertonic stress (Figures 4A and B).

## Discussion

The role of PGE_2_ in cancer development is usually described as an autocrine factor capable of modulating many aspects of the cancer cell biology, in particular those of epithelial origin [28], [29]. PGE_2_ has been shown to increase proliferation, metastatic capacity and production of pro-angiogenic factors [17], [30], [31]. However, such studies usually lack information on the stimuli that will drive the arachidonic acid cascade and ultimately PGE_2_ generation beyond induced expression of COX-2. We have recently demonstrated that a hyperosmotic milieu can induce COX-2 expression and PGE_2_ generation in Caco-2 colon cancer cells [24]. Most importantly, hyperosmolarity can trigger the limiting step in PGE_2_ generation by activating cPLA_2_-α and inducing the release of free arachidonic acid. Normal intestinal epithelial cells showed no production of PGE_2_ under the same hyperosmotic stimulus. Having identified a relevant physiological stimulus for colon cancer cells, we investigated the effect of hypertonic medium on the production of the major pro-angiogenic factor, VEGF, and the potential autocrine influence of PGE_2_.

Exposure of Caco-2 cells to hypertonic medium led to a significant production of VEGF. As demonstrated before this VEGF production occurred in parallel with the PGE_2_ generation. PGE_2_ is described as a potent inducer of VEGF based on experiments where COX-2 overexpression increased VEGF production by colon and breast cancer cell lines. Furthermore, this VEGF production was inhibited by selective COX-2 inhibitors. We therefore sought to investigate the autocrine influence of PGE_2_ generation on Caco-2 cells stimulated by hypertonic medium. Suprisingly, inhibition of either cPLA_2_ or COX-2, by ATK or NS-398 respectively, further increased the production of VEGF. This effect could be attributed to inhibition of PGE_2_ generation as restoration of PGE_2_ levels by exogenous addition reverted VEGF production to its original levels in ATK treated Caco-2 cells. HCT116 cells can also be activated by hypertonic medium to produce VEGF. However, HCT116 cells neither express COX-2 nor produce PGE_2_ [31] despite the cells being activated or not. Thus, the lack of effect of ATK and NS-398 on HCT116 excludes any potential off target effects of these inhibitors [32].

PGE_2_ acts on a group of G-protein-coupled receptors (GPCRs). There are four GPCRs responding to PGE_2_ designated subtypes EP1, EP2, EP3 and EP4, leading to distinct signaling pathway and overall biological effect [27]. To determine the dependence of PGE_2_ autocrine effects on EP signaling, we used a nonspecific antagonist of all four EP receptors, AH 6809. The treatment with AH 6809 mimicked the effect of inhibition of VEGF production by PGE_2_, indicating that PGE_2_ signals through its plasma membrane receptors to down regulate VEGF production induced by hypertonic medium. The particular subtype involved appears to be EP2 as its selective agonist, Butaprost, is able to fully substitute for PGE_2_. On the opposite, neither EP1 nor EP3 agonists treatments presented the inhibitory effect on VEGF production. EP2 seems to couple with increased cAMP levels and subsequent activation of PKA, as inhibition of PKA by H-89 reproduces the effects of inhibiting PGE_2_ generation or action. It is important to note that the experiments were performed with PGE_2_ and its analogs in concentrations that were compatible with the endogenously produced levels. Effects in VEGF production by colon cancer cells have been ascribed to PGE_2_ using concentrations of up to 100 µM [13] what far exceed the amount of PGE_2_ actually needed to activate its receptors [27] or what is produced in the tumoral mass [33].

It has been shown the activation of EP2-cAMP-PKA-GSK-3 signaling pathway leads to decrease of beta-catenin phosphorylation, allowing its translocation and activation of Tcf/Lef dependent-transcription (for a review, see [34]) of genes involved in cancer, such as COX-2 and VEGF. However, in our model of hypertonic stress, EP2 signaling pathway activation causes repression of VEGF production. To better understand the regulation of this pathway in the hypertonic stress we investigated the role of GSK-3in this activation with the use of SB216763, a competitive GSK-3 α and β inhibitor (50–5000 nM, data not shown). The treatment increased the VEGF production, indicating that the inhibitory effect of EP2 on the angiogenic factor production is not dependent on this kinase. One possibility for the distinct effect of EP2 activation in hypertonic stress is that this regulation is occurring through cAMP. Some studies have been shown cAMP can inhibit the production of cytokines by inhibiting Ras-dependent signals by PKA, inactivating MEK/ERK signaling or by blocking phosphorylation of p38 MAPK [35]–[37]. As ERK/p38 MAPK pathway is involved in our model inducing VEGF production, such findings could be indicative of the mechanism by which EP2 signaling is blocking VEGF production in hypertonic stress.

To determine whether the inhibitory role of PGE_2_ may be extended to other stimuli, we used CoCl_2_ to induce HIF-1α stabilization and mimick the response to hypoxia [13]. As shown for hypertonic stimulation, CoCl_2_ induced PGE_2_ generation was dependent on COX-2. The PGE_2_ generation was also paralleled by VEGF production. Induction of VEGF production by CoCl_2_ has been shown before in human fibroblasts [38], lung cancer cell line [39], retina epithelium [40], glioma cell lines [41], prostate cancer cell lines [42] and in astrocytes [43], however there was no attempt to investigate the potential production of PGE_2_ or its autocrine effects. Inhibition of COX-2 inhibited VEGF production induced by CoCl_2_, indicating a stimulatory role for PGE_2_ and that the autocrine effect of PGE_2_ is dependent on the type of stimuli used.

Hypertonic medium induces activation of several MAP kinases that may be involved in regulating VEGF expression [24]. Since these MAP kinases are also involved in stimulating cPLA_2_-α activity and production of PGE_2_, we eliminated the inhibitory effect of PGE_2_ before attempting to analyse their role on VEGF production. Caco-2 cells activated by hypertonic medium in the presence of NS-398 clearly show a marked dependence on p38 and less so on ERK 1/2 to produce VEGF. CoCl_2_-induced VEGF production by Caco-2 cells showed similar sensitivity profile to MAP kinase inhibitors with the exception of a marked reduction by the JNK inhibitor. The distinct roles for JNK pathway indicate that differences in hypertonic medium and CoCl_2_-induced production of VEGF go beyond sensitivity to autocrine inhibitory effects of PGE_2_. It is currently under investigation if such signaling differences may be responsible for the distinct effects of PGE_2_ on each type of stimuli.

One of the hallmarks in the current model for the regulation of angiogenesis in the tumor mass, particularly in colon cancer, is the regulation of endothelial cell function by the cancerous cells. Accordingly, the role of PGE_2_ in angiogenesis is limited to an autocrine stimulation of pro-angiogenic factors production by the tumor cell. Although interesting, this model neither comprises the potential external stimuli involved in COX-2 expression and VEGF production nor situations where the cell expressing COX-2 and producing PGE_2_ is other than the cancer cell. For instance, ectopic growth of HCT116 and HT29, cells that do not express COX-2 or produce PGE_2_, is dependent on COX-2 expression by endothelial and stromal cells [44]. COX-2 expression in mouse models of familial adenomatous polyposis, *Min* [33], [44] and Apc^Δ716^ [45] mice, is restricted to stromal and interstitial cells. Differences in JNK dependency and autocrine PGE_2_ inhibitory effect on VEGF production by the same colon cancer cell line are a clear indication on how the model must also take into account that these cells are exposed to different microenvironmental stimuli.
